# Navigating political dilemmas: collaborative efforts between a health trust and municipalities, before and during the COVID-19 pandemic in Norway

**DOI:** 10.1108/JHOM-02-2025-0093

**Published:** 2026-01-07

**Authors:** Lena Bjørge Waage, Roar Eilev Amdam

**Affiliations:** Volda University College, Volda, Norway

**Keywords:** Health services, Multi-actor collaboration, Instrumental and communicative rationality

## Abstract

**Purpose:**

The Norwegian Government expects collaboration between health trusts and municipalities to promote sustainable, coordinated health services. The coordination reform (2012) introduced various instruments. This article analyses how these instruments created dilemmas, affecting collaboration differently before and during the COVID-19 pandemic.

**Design/methodology/approach:**

This article applies a comparative qualitative case study design with a theoretical interpretative focus. The material includes public documents, cooperation agreement and individual interviews covering experiences from formal collaboration structures before the pandemic and more informal ad hoc collaboration during the pandemic.

**Findings:**

Before the pandemic, collaboration involved power imbalances and discussions over resources and financing. During the pandemic, actors appeared equal and focused on tasks and solutions. This article highlights how a better balance between instrumental and communicative rationalities supported this change.

**Practical implications:**

The findings suggest that collaboration is more effective when policy design supports both instrumental clarity and communicative engagement. Clear goals, mutual recognition of local perspectives and flexible structures, where relevant actors are entrusted with decision-making authority, can strengthen policy support and enable local mobilization.

**Originality/value:**

This article illustrates how collaboration between a health trust and municipalities represents both multi-actor collaboration and multi-level governance. The findings contribute to the academic discussion on how balancing hierarchical control with communicative approaches may foster collaborative outcomes. They underscore the need for context-sensitive governance approaches that may support adaptive and sustainable collaboration across diverse policy areas.

## Introduction

Organization and management of public health services have long been key issues for the Nordic countries, which are reflected in several health reforms (Kirchhoff *et al.*, 2019; Vik and Hansson, 2024). These reforms have aimed to address challenges related to rising costs and capacity, leading to a shift from new public management-inspired market mechanisms to an emphasis on holistic patient care and health promotion through collaboration (Hanssen *et al.*, 2014; Martinussen and Magnussen, 2009). However, successful collaboration in the health sector remains difficult to realize in practice due to a variety of factors (Auschra, 2018), such as conflicting interests and priorities (Abbas *et al.*, 2022; Axelsson and Axelsson, 2006; Ribeiro *et al.*, 2019), imbalance in power and knowledge (Steihaug *et al.*, 2016; Rodríguez *et al.*, 2007) and financial, organizational and bureaucratic barriers (Lluch, 2011; Jørgensen *et al.*, 2024; Magnussen, 2023). These impediments can foster resistance to collaboration and exacerbate tensions in the modern healthcare system (Vik and Hjelseth, 2022). Why collaboration continues to be elusive is still an open question, underscoring the need for studies that probe more deeply into the dynamics of collaborative efforts. There is a growing call for research that examines collaboration across diverse contexts, recognizing that contextual factors can influence the capacity to implement health policy (Breimo and Anvik, 2022; Cairney *et al.*, 2021; Emerson and Nabatchi, 2015).

Building on a document analysis of collaborative practices between a health trust and municipalities in a Norwegian county (Waage and Amdam, 2025), this article incorporates interview data to provide a more nuanced understanding of how political dilemmas are navigated across two distinct settings: the “normal situation”, following the coordination reform (Norwegian Ministry of Health and Care Services, 2009) and crisis collaboration during the COVID-19 pandemic. Whereas collaboration in the normal situation relied on statutory and formal structures (Norwegian Ministry of Health and Care Services, 2011a; Møre and Romsdal Health Trust, 2015), collaboration during COVID-19 was shaped by national government management (Christensen *et al.*, 2023) and informal practices (Møre and Romsdal Health Trust, 2020). This demonstrates how crises may necessitate more adaptive forms of collaboration, with potential positive outcomes including reduced conflict, strengthened trust and innovations (Amdam *et al.*, 2025; Parker *et al.*, 2020; Roberts, 2000).

It is well documented that a pandemic, as a global societal crisis (Remuzzi and Remuzzi, 2020), can foster effective collaboration (Christensen *et al.*, 2016; Christensen and Ma, 2021; Moynihan, 2008). The pandemic has been widely studied both globally and nationally in Norway (Capano *et al.*, 2020; Christensen and Lægreid, 2020; Christensen *et al.*, 2023; Lynggaard *et al.*, 2023; NOU, 2021:6, 2022:5; 2023:16; Remuzzi and Remuzzi, 2020). However, fewer studies have focused on regional and local responses (Braut, 2023; Smoke *et al.*, 2023). Traditionally, research has emphasized collaboration within formal frameworks (Isett *et al.*, 2011) while also acknowledging the importance of informal arrangements (Agranoff, 2006). More informal collaborations are often established to meet acute needs for coordination across sectors (Parker *et al.*, 2020). This article contributes by analysing collaboration across both formal and informal settings. Existing research on health collaboration has largely highlighted the service perspective (Røiseland, 2023; Tveit Sandvin *et al.*, 2022). By adopting a multi-actor and systems perspective, this article provides a qualitative complement to the quantitative approaches that currently dominate this research field (Gremyr *et al.*, 2024; Rommetvedt *et al.*, 2025).

Furthermore, this article addresses part of the need for conceptual grounding (Auschra, 2018), as health research on collaboration is often characterized by overlapping terminology and converging concepts (Abbas *et al.*, 2022; Axelsson and Axelsson, 2006). More broadly, the collaboration literature is marked by complexity and fragmentation (Ansell and Torfing, 2016; McGuire, 2006; Pierre and Peters, 2020). Clarifying the conceptual and theoretical foundations can strengthen the potential for cumulative knowledge and carries important implications (Isett *et al.*, 2011; Røiseland and Lo, 2019).

Anchored in the discourse of collaboration governance (Ansell and Gash, 2008) and collaborative planning theory (Davoudi, 2015; Healey, 1997; Innes and Booher, 2015), this article argues that collaborative patterns can be understood through the interplay between instrumental and communicative rationality. Instrumental rationality entails top-down control, rule-based governance and efficiency, framing individuals as self-interested actors requiring regulation. Communicative rationality, by contrast, emphasizes bottom-up processes, democratic engagement and shared meaning-making, portraying individuals as capable of pursuing both personal and collective goals.

Collaboration as a governance strategy in health reforms reflects a communicative turn in the public governance discourse (Amdam, 2025). Traditionally, instrumental rationality has dominated modern social development (Habermas, 1989, 1996). The resulting tension between instrumental and communicative logics may create dilemmas for collaboration, but they also open up spaces of opportunity. When balanced, these logics can provide favourable conditions for power relations and shape the action patterns of social actors. In such contexts, interests and values become central, and the more strongly they are emphasized, the less reliance there is on coercion (Castell, 2009; Innes and Booher, 2015).

This article answers the following research questions:

RQ1.
What characterizes multi-actor collaboration between a health trust and its associated municipalities before and during the COVID-19 pandemic?

RQ2.
How can we understand these characteristics considering well-known dilemmas linked to instrumental and communicative rationalities?

The next section elaborates on the Norwegian context, followed by a presentation of the theoretical framework. The subsequent sections outline the research design, data foundation and analysis strategy. Results and discussion are integrated, with findings examined through the theoretical lens. The conclusion summarizes the main findings and highlights key implications.

## Collaboration as a tool in Norwegian health policy

The overall goal of Norwegian health policy is to strengthen the ability of health trusts and municipalities to collaborate as equal partners, ensuring that no single actor's premises dominate the partnership. Since neither party possesses complete expertise, integrating their professional competencies is essential for an effective distribution of responsibilities, promoting coordinated and resource-efficient care (NOU, 2005:3; Norwegian Ministry of Health and Care Services, 2009, 2019, 2024a, b).

However, fulfilling these expectations has been challenging, partly because the actors operate under separate legal frameworks (Norwegian Ministry of Health and Care Services, 1999, 2011a), with dual responsibility for public health services. In the Norwegian governance model, municipalities handle primary health care, while the state, through regional and local health trusts, manages specialist health services (Saunes *et al.*, 2024). Different financing mechanisms may also cause costs of joint measures falling on one level, while benefits arise at another (Magnussen, 2023; Nylenna, 2024). Inspired by the “National Health Service” (NHS) models in the UK, New Zealand and Southern Europe, which emphasize centralization, the Norwegian management model emphasizes decentralization. This is a distinctive feature of the Nordic health systems. While Sweden and Denmark have concentrated responsibility at one level (county and regional level), Norway and Finland have divided responsibility for public health services between municipalities and hospitals (Martinussen and Magnussen, 2009). This division can be understood as co-governance (Røiseland and Vabo, 2012) and multi-level governance (Bache *et al.*, 2016; Helgøj and Aars, 2008; Rommetvedt *et al.*, 2014).

To strengthen the collaboration between health trusts and municipalities, the Norwegian Government launched the coordination reform in 2012. The reform employs various instruments, such as financial, structural, regulatory and normative measures (Norwegian Ministry of Health and Care Services, 2009). Two key initiatives are statutory cooperation agreements (Norwegian Ministry of Health and Care Services, 2011a) and municipal payment obligations for patients ready for discharge (Norwegian Ministry of Health and Care Services, 2011b). Both can be associated with an international Western trend from the 2000s, aiming to achieve better coordination and to ensure that health services are delivered more cost-effectively at the municipal level (Gautun *et al.*, 2016; Grimsmo *et al.*, 2015; Nødland and Rommetvedt, 2019; The Research Council of Norway, 2016).

The cooperation agreements are anchored in a legally mandated system of agreements (Norwegian Ministry of Health and Care Services, 1999, § 2-1e; 2011a, § 6–1), building on earlier voluntary agreements with the Norwegian Association of Local and Regional Authorities (KS). The aim has been to clarify responsibilities within 11 (today 13) specified areas (including admission, discharge and prevention), establish frameworks for collaboration and ensure that health trusts fulfil their obligations to municipalities, such as providing necessary expertise to support safe and effective service delivery (Norwegian Ministry of Health and Care Services, 2009, p. 77, 2011a, § 6–2). As part of the financial incentive schemes, municipalities were given co-financing responsibilities for somatic specialist health services provided to their residents (discontinued January 1, 2015), as well as payment obligations for patients who were medically ready for discharge but remained in hospital (Norwegian Ministry of Health and Care Services, 2011a, 2014).

Even though the cooperation agreements have been important for clarifying task allocation and responsibilities, they have not led to notable changes regarding equality between the health trusts and municipalities (Brekk and Kirchhoff, 2015; Rommetvedt, 2021; Rommetvedt and Nødland, 2020; The Research Council of Norway, 2016; Williksen *et al.*, 2014). The payment obligation has contributed to shorter hospital stays for selected patient groups (Melberg and Hagen, 2016), but it has also led to a stronger focus on finances and increased conflict in collaboration (Gautun *et al.*, 2016; Martens and Veenstra, 2015; Melberg and Hagen, 2016; The Research Council of Norway, 2016; Waage and Amdam, 2025). To promote partnership thinking, Norwegian authorities launched a new concept, *Healthcare* *Communities*, in October 2019 (Norwegian Ministry of Health and Care Services, 2019). A status-mapping study found that collaboration remains challenging due to legal and economic factors, as well as separate management lines (Rambøll Management Consulting, 2024).

## Theoretical framework

To explore collaboration between a health trust and its associated municipalities across two distinct contexts, we apply two complementary theoretical perspectives: collaborative governance and collaborative planning theory. While collaborative governance provides an institutional lens for examining formal decision-making processes, collaborative planning theory emphasizes learning, communication and the co-construction of meaning. By incorporating both instrumental and communicative rationalities, these perspectives allow for a more nuanced understanding of how collaboration unfolds in practice.

Collaborative strategies complement hierarchical and market-based systems, offering potential for innovation (Torfing, 2019). Central concepts include interdependence, self-organization and autonomy from the state, where multiple processes inform the political system (Osborne, 2010; Rhodes, 1997). One positive outcome is social learning, in which different actors inform each other's actions through mutual knowledge exchange. Over time, such interaction can evolve into a self-organizing, adaptive system capable of addressing complex societal challenges (Roberts, 2000). Power, governance and democracy are shaped through communication (Selg and Ventsel, 2020).

Although successful institutional collaboration can be developed (Ansell and Gash, 2008), it can be constrained by what actors can realistically contribute in terms of resources (Seo, 2025). Collaborative alliances are rarely equal and are often shaped by power asymmetries, leading to uneven and contested value distribution (Osborne, 2010). To incentivize collaboration, authorities may introduce rewards or sanctions through legislation, illustrating how collaboration operates in the shadow of hierarchy (Héritier and Lehmkuhl, 2008). In times of crisis, this interplay may shift, with increased hierarchical command-and-control governance, with expert knowledge playing a more prominent role in decision-making (Christensen *et al.*, 2023; Hungnes *et al.*, 2022).

The literature reveals a diversity of theoretical approaches to examining multi-actor collaboration (Ansell and Torfing, 2016; Bianchi *et al.*, 2021; McGuire, 2006; Pierre and Peters, 2020), including new public governance (Osborne, 2010), policy networks (Isett *et al.*, 2011; Kickert *et al.*, 1997), partnerships (Pierre, 1998; Røiseland, 2013; Sullivan and Skelcher, 2017), co-governance (Kooiman, 2003; Røiseland and Vabo, 2012) and co-creation (Røiseland and Lo, 2019; Torfing *et al.*, 2019).

In this study, we draw on collaborative governance, which can be defined as:

A governing arrangement where one or more public agencies directly engage non-state stakeholders in a collective decision-making process that is formal, consensus-oriented, and deliberative and that aims to make or implement public policy or manage public programs or assets (Ansell and Gash, 2008, p. 544).

This approach aligns with Norwegian health policy, where health trusts, municipalities and service users are expected to reach shared agreements that promote coherence and sustainable health services (Norwegian Ministry of Health and Care Services, 2009, 2019, 2024a, b).

To examine how this ambition is adapted to and implemented through collaborative processes, we apply collaborative planning theory, in which collaboration is understood as a continuous learning process where factual knowledge is integrated with practical skills and moral judgement, ultimately put into action. Through such processes, collaboration contributes to capacity-building and enhances the ability to address complex societal challenges (Davoudi, 2015).

Rooted in pragmatism (Allmendinger, 2009), collaborative planning theory integrates instrumental and communicative rationality, emphasizing the dynamic interaction between actors and structures (Healey, 1997). From an instrumental perspective, actors act in compliance with laws and regulations, guided by evidence-based knowledge. In contrast, the communicative perspective emphasizes democratic opinion formation, where communicative power can be translated into action, and political power provides legitimacy for structural change (Amdam, 2020, 2024). The coordination reform reflects this interplay by introducing legal requirements for collaboration while also encouraging equal dialogue and joint problem-solving.

Emerging in the 1980s and 1990s, the communicative approach, drawing on Habermas (1989, 1996), views communication as socially constructed action. Multi-actor collaboration, when situated in context, time and place, along with informed judgement, may support tailored responses to complex issues (Innes and Booher, 2015, p. 4). However, proponents of instrumental planning, with a more positivist stance, criticize this approach for its idealism and inefficiency. They argue that interests are fixed, values predetermined and that top-down planning is more effective. Much of the critique targets the assumption of equal dialogue and the feasibility of consensus. Yet, the goal of communicative planning theory is not to uncover absolute truths but to find practical solutions to shared problems (Innes and Booher, 2015, pp. 4–5). Instrumental approaches suit predictable processes focused on rules and facts to enhance efficiency, while communicative approaches are better suited to unpredictable situations, emphasizing deliberation, service quality, democracy and justice (Davoudi, 2015).

Tensions between instrumental and communicative rationalities are also evident in the health sector. Instrumental logic emphasizes disease treatment and prevention through top-down control, while communicative logic promotes health and equity by building local capacity and health literacy (Laverack and Labonte, 2000). Policymaking is often framed as a linear, technical process (Godziewski, 2020, p. 2), but in practice, it is shaped by both rationalities and is challenged by power imbalances (Cairney, 2023; Cairney *et al.*, 2021).

Although instrumental and communicative approaches may seem incompatible, Innes and Booher (2015) argue that they can be complementary. Drawing on Castells' (2009) theory of communicative power, they have identified four key tensions: community knowledge (lay knowledge) versus science (expert knowledge), collaboration versus conflict, process versus outcome and communication power versus state power. A central argument is that communication can drive collaboration by shaping shared understanding and guiding collective action. Although power is often associated with coercion, it can also be constructed through dialogue, reducing the need for dominance.

It is important to emphasize that Innes and Booher's dichotomies should not be viewed as mutually exclusive. Rather, the conditions that shape collaboration influence one another across the different dimensions.

### Community knowledge versus science

The dichotomy contrasts the instrumental approach, emphasizing scientific expertise, with the communicative approach, valuing diverse expertise and shared meaning-making. Instrumental logic is well integrated into education and institutions, often marginalizing lay and community knowledge. Innes and Booher (2015) argue that it is counterproductive to oppose “soft” qualitative knowledge against “hard” scientific data. Combining different knowledge sources in a common learning process yields more accurate and meaningful insights.

### Collaboration versus conflict

Innes and Booher (2015) contend collaboration often emerges from conflict, rather than being its opposite. Consensus is not always the goal, since differing actors can agree to disagree and still collaborate. Collaboration can be conflict-ridden, as participants represent groups affected by collaborative decisions. Instrumental logic promotes self-interest and hierarchical decision-making, while communicative logic fosters altruism, interdependence and mutual learning.

### Process versus outcome

Innes and Booher (2015) assert that the distinction between processes and outcomes is false, arguing that participants engage in processes because they care about outcomes. The process influences the outcome, as participants who feel heard and treated fairly are more likely to support it. Instrumental logic focuses on procedures and results, while communicative logic emphasizes democratic, inclusive processes and mutual learning (Habermas, 1996).

### Communication power versus state power

Innes and Booher (2015) argue that power is shaped through communication, influencing rules, norms and practices. Public, private and civil society actors all contribute to structuring society. At the core of democracy lies the principle that legitimacy derives from the people. While instrumental logic justifies power through top-down control, communicative logic emphasizes bottom-up processes, where individuals are seen as capable of balancing their own and others' goals. Through dialogue, local communicative power can reshape institutions by altering shared understandings.

## Method

### Design

To illuminate collaborative patterns between a health trust and associated municipalities across distinct contexts, we used a comparative qualitative case study design with a theoretical interpretative focus (Bukve, 2021). This approach enables an in-depth study of the collaboration in the two different contexts, which may also provide useful insights for understanding collaboration in other contexts (Gerring, 2004). The context in which practices occur is an essential element of case studies to provide understanding and learning (Flyvbjerg, 2006).

In this study, the contexts for exploring the collaboration are specifically the period before the coronavirus pandemic (*2016–mid-2021*) (case 1) and during the coronavirus pandemic (*March 2020–mid-2021*) (case 2). By using a comparative case study approach, this design allows for comparison across axes: horizontally, by comparing how similar policies or phenomena unfold in connected and socially produced locations; vertically, which traces phenomena across scales; and transversally, by tracing phenomena and cases over time (Bartlett and Vavrus, 2017, p. 14).

The case design enables a holistic perspective, conceptualizing multi-actor collaboration as a complex system of interdependent components (Bukve, 2021, p. 85), involving both structural and procedural aspects within a specific context. This aligns with a process-oriented approach that seeks to uncover meaning in phenomena through the comparison of human experiences, situations, events and the processes that connect them (Bartlett and Vavrus, 2017, pp. 6, 8). In this study, theory has been used to interpret patterns in the empirical material and to highlight factors that influence collaboration (Bukve, 2021, pp. 91–92). We combine inductive and deductive reasoning (Nilsen, 2015), meaning that no specific theory guided the data collection. Instead, theoretical perspectives were selected based on the insights emerging from the data and subsequent analysis.

### Data

The empirical basis includes 13 key informant interviews from the collaboration between Møre and Romsdal Health Trust and municipalities in Møre and Romsdal county. Collaboration between these actors also constitutes a case in a research project exploring collaboration before, during and after the COVID-19 pandemic (Amdam *et al.*, 2025). As part of this research project, political documents, cooperation agreements and meeting minutes from both contexts have been reviewed and supplement the data material in this study (see Waage and Amdam, 2025, p. 108). This has provided a rich body of material, which is a strength of qualitative data triangulation (Flick, 2004). The documents were sourced from the Norwegian Government and the health trust's websites.

**Figure 1:**
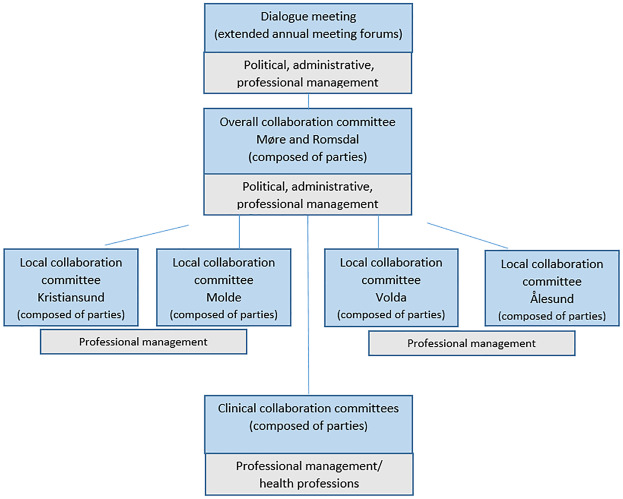
Collaboration structure between the health trust and the municipalities in Møre and Romsdal 2016-1st half of 2021 (“before the pandemic”) (Møre and Romsdal Health Trust, 2015). Source: Authors’ own work

**Figure 2:**
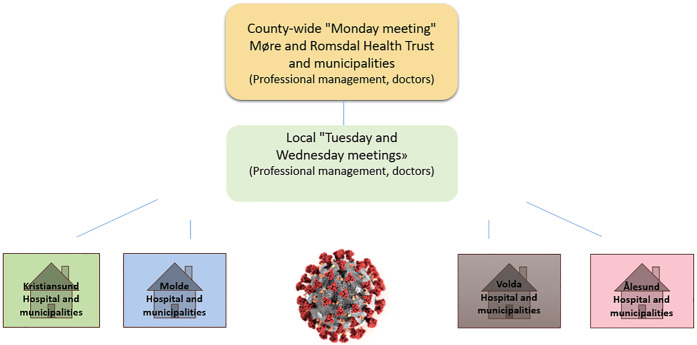
Collaboration structure between the health trust and the municipalities in Møre and Romsdal during the corona pandemic (Møre and Romsdal Health Trust, 2020). Source: Authors’ own work

The health trust encompasses hospitals in Kristiansund, Molde, Volda and Ålesund [Aalesund]. In the spring of 2025, the hospitals in Kristiansund and Molde were merged into the new Nordmøre and Romsdal Hospital (Sjukehuset Nordmøre og Romsdal). The health trust provides specialist health services to approximately 270,000 residents in the county's 27 municipalities. To explore these actors' collaboration, we have taken as our starting point the collaboration that took place within a formal collaboration structure before the pandemic (Figure 1) (case 1) and an informal, ad hoc collaboration structure during the pandemic (Figure 2) (case 2). To enable comparisons, we have focused on collaboration at the county-wide and local levels related to the four hospital areas. This means that clinical collaboration committees have not been included in the scope of this study.

### Selection and interview process

The interview data are based on individual semi-structured in-depth interviews (Kvale and Brinkmann, 2009), with a strategic selection of informants (Malterud, 2013). This included 13 key informants (6 men and 7 women) (Table 1). Inclusion criteria were based on the informant's experiences from the collaboration in the two different contexts, representing various perspectives, diversity in roles, organizational affiliation and geographical distribution.

**Table 1:** Overview of informants

Organization	Informants' role/function
Møre and Romsdal Health Trust	Clinical manager (2)
	Department manager (1)
	Adviser (1)
Municipalities in Møre and Romsdal	Mayor (1)
	Municipal Director (1)
	Head of Municipal affairs (3)
	Municipal Medical Officer (1)
Others	County Governor's office (1)
	General practitioner (GP) (1) (*also affiliated with the health trust*)
	User representative (1)

**Source(s):** Authors’ own work

Although the data selection is weighted toward municipal sources, this does not compromise the validity of the analysis. This is justified through a case-centred analytical strategy (Bukve, 2021) and a strategically composed dataset designed to best illuminate the research questions (Malterud, 2013, p. 54).

The interviews were guided by the following themes: collaboration, the coordination reform, cooperation agreements, collaboration structure, collaboration during the pandemic and learning/development. To ensure relevance and structure, we conducted a test interview. No changes were deemed necessary. The interviews were conducted in October and November 2021, including physical attendance (9) and digitally via Teams (4), with a duration of 1–1.5 h per interview. The first author was the moderator for all interviews, and a co-moderator also participated in 9 of the total 13 interviews. The purpose was to reduce potential bias (Malterud, 2013), as well as interview effect, based on the first author's experience from the collaboration and knowledge of the informants.

### Analysis and findings

The analysis strategy combines inductive and deductive reasoning, building on a social constructivist approach (Esmark *et al.*, 2005). This means that we have sought to give meaning to collaboration through conceptualizing and examining the relationship between enabling and limiting structures and the actors involved in the collaboration.

The inductive approach is inspired by Malterud's (2013) systematic text condensation method, based on Giorgi's phenomenological analysis strategy (Giorgi, 1985, 2009). We combined this with theoretical interpretation (Bukve, 2021), which involved evaluating relevant theories through conceptual testing (Tjora, 2023) and posing questions to explore underlying meanings in the data. The purpose was to understand influencing factors of the collaborative outcomes (Nilsen, 2015, p. 2). The process involved working through various stages in a non-linear manner, with a high degree of interaction between different steps. We used Microsoft Word to maintain structure and overview.

In step 1, each author reviewed the transcribed interviews to form an overall impression. To gain an overview, the first author structured relevant data under thematic headings from the interview guide. The text excerpts were reviewed and validated by the second author.

In step 2, we undertook a more systematic review of the data material, still closely following the informants’ descriptions with the aim of identifying meaningful units. To aid the process, we structured excerpts from the transcribed data material from each informant in a Word table. The meaningful units at this level were both short and long sentences, as well as paragraphs. In a second column, we also noted initial codes to systematize the content while reflecting on common features in the data set. These common features included, for example, codes related to questions of equality, financial mechanisms and resource barriers, which seemed to challenge the ability to reach shared understanding. The codes reflecting these common features formed the basis for categories.

In step 3, we summarized the findings by presenting them in a written report. Table 2 outlines the key points. The analysis led us to conclude that collaboration varies across the two contexts, highlighting dilemma areas related to equality and the ability to achieve consensus-based task distribution. Both are fundamental components for realizing the goals of improved coherence and efficient resource utilization in healthcare services (Norwegian Ministry of Health and Care Services, 2009, 2019, 2024b).

**Table 2:** Summary of main findings

Theme/Dilemma-area	Formal collaboration (pre-covid-19) (case 1)	Informal collaboration (during Covid-19) (case 2)
Fulfilling the expectation of equality	Main findings Striving for equality, but the health trust is perceived as superior to the municipalities Municipalities need to come together in drafting agreements The health trust act as a premise setter in discharge situations Emphasis on medical professional discourse can have an exclusionary effect in collaborative meetings	Main findings The health trust and municipalities appear equal The health trust and municipalities recognize that they are mutually dependent on each other's expertise and contributions Emphasis on knowledge diversity and dialogue has an inclusive effect in collaborative meetings
Fulfilling the expectation of task sharing through consensus-based dialogue	Main findings Striving for division of tasks, but challenged by the ability to engage in consensus-based dialogue Planning services in municipalities is challenged by limited resources (competence, finances) Municipal payment obligation for patients ready for discharge has contributed to conflicts Different levels of administration and financing constitute a barrier, contributing to resource-related discussions and hindering progress in joint projects The individual actor's autonomy challenges decision-making authority and collaboration	Main findings Enables task sharing through consensus-based dialogue Discussions were concise and to the point, solution-oriented, task-focused and practical The cooperation agreement and formal structure became less important A new informal professional representative collaboration structure contributed to more effective decision-making processes

**Source(s):** Authors’ own work

In step 4, we evaluated various theoretical frameworks to interpret the findings, which revealed clear variations in collaboration patterns before and during the COVID-19 pandemic. A central criterion was the framework's ability to shed light on what influences actors' perceptions and actions and how these shape collaboration. We found theories of rationality and the dichotomies identified by Innes and Booher (2015) well suited to making sense of collaborative behaviour before and during the COVID-19 pandemic. For example, instrumental self-interest provided fertile ground for discussions and conflicts prior to the pandemic, whereas during the pandemic actors recognized their mutual dependence, which supported communicative values such as equality and shared meaning-making. Although the framework offers insight into key tensions that might affect collaboration, other frameworks may provide alternative theoretical explanations. We argue, however, that Innes and Booher's approach helps shed light on how contextual conditions influence which forms of rationality are emphasized, with implications for policy and practice alike.

### Ethical considerations

We contacted the informants by email and telephone, attaching an information letter and consent form. The informants have approved the quotes used in this study. This study has been submitted to the Regional Committees for Medical and Healthcare Research Ethics (REK, 2021). It was not found to be subject to submission. This study has also been presented and approved by the Data Access Committee of the Norwegian Medical Association (DAC, 2021). We have recorded, transcribed and stored the interviews in accordance with research ethics guidelines (NESH, 2023).

### Declaration of generative AI

During the preparation of this work, the authors used Copilot to check grammar and spelling and improve readability and language. After using this tool, the authors reviewed and edited the content as needed and took full responsibility for the final version of the manuscript.

## Results and discussion

Through equal collaboration and consensus-based division of responsibilities, Norwegian authorities expect health trusts and municipalities to develop coherent and sustainable health and care services (NOU, 2005:3; Norwegian Ministry of Health and Care Services, 2009, 2019, 2024a, b). Our findings show that the implementation of these expectations differed before and during the COVID-19 pandemic. The following sections present the findings through Innes and Booher's (2015) four dichotomies, focusing on the tension between instrumental and communicative rationality.

### Managing equality in the tension between community knowledge versus science

The expectation of equal partnership is based on the understanding that neither health trusts nor municipalities possess complete expertise and that effective division of responsibilities requires the integration of their respective competencies (NOU, 2005:3, p. 15; Norwegian Ministry of Health and Care Services, 2009, 2019). This principle is reflected in the cooperation agreement: “The collaboration is built on trust and openness between equal parties who are mutually dependent on each other in order to provide a good comprehensive service” (Møre and Romsdal Health Trust, 2015, p. 5). However, municipal informants described that the health trust often appeared dominant, for example, in the drafting of agreements prior to the pandemic. The municipalities therefore began coordinating their input, which contributed to a more balanced dialogue.

Power asymmetries could also arise in discharge processes, where the health trust could define which services the municipalities were expected to provide. One municipal leader noted the difficulty of challenging a hospital specialist. The health trust has attempted to raise awareness of municipal autonomy, but cultural change is slow.

Power imbalances also emerged in collaboration meetings, often due to differing institutional roles and professional language. The mayor described the dialogue meetings (the highest political and/or administrative level within the formal collaboration structure) as dominated by “tribal language”, feeling like an outsider. Such imbalances can hinder the openness necessary for developing collective solutions. The quote below illustrates this:

One does not like to be told things […] it only takes a few crooked words, and you might get a little defensive or a little critical. Then you don't have the openness to want to get the best solution, then you're already a little on the warpath.

(User representative)

During the pandemic, the expectation of equality was not questioned. As the general practitioner (GP) noted, there was a sudden shift in the mindset: “Suddenly municipalities and the health trust discovered that they had to collaborate”. This shift was accompanied by a dialogue marked by mutual appreciation and recognition of each other's contributions and expertise. As a municipal medical officer reflected:

I feel we have gained a higher status and GPs see how important specialties like microbiology are. […] now we see how important all parts of the health service are. […] I think we have learned from each other how important it is to collaborate, to utilize each other's expertise and viewpoint.

(Municipal Medical Officer)

A hospital manager praised the municipalities' efforts: “Without such a well-functioning municipal health service, we would have been overrun by patients in the hospitals. Municipalities have done a great job, and we are simply impressed”.

The findings show how the expectation of acting as equal partners manifested differently before and during the COVID-19 pandemic.

Before the pandemic, power dynamics were shaped by instrumental logic's emphasis on scientific expertise. In this context, the health trust, acting as a “specialist”, was granted greater influence and often adopted a dominant position toward municipalities, which functioned more as “generalists” with broader, context-sensitive knowledge. This imbalance is exemplified by the municipalities’ need for collective action during contract negotiations, the health trust's tendency to set conditions in discharge situations and the politician's challenge in being recognized as an equal partner in collaborative meetings. Given that knowledge produces power (Innes and Booher, 2015), this hierarchy undermines the ambition of equal partnership.

During the pandemic, a broader range of knowledge was more openly acknowledged. Collaborative meetings increasingly included medical doctors from both the health trust and the municipalities in leading roles. Although they represented different roles and areas of expertise, they recognized the value of complementarity as essential to managing the crisis and to developing context-sensitive local solutions. According to Innes and Booher (2015, p. 8), such mutual recognition aligns with communicative rationality, where shared understanding, interpretation and critical reflection enable joint action.

### Managing economic instruments in the tension between collaboration versus conflict

The coordination reform was accompanied by a range of policy instruments (Norwegian Ministry of Health and Care Services, 2009), including municipal payment obligation for patients medically ready for discharge but who remain hospitalized (Norwegian Ministry of Health and Care Services, 2011b). Although this regulation provides guidance, it can also be adapted and anchored in local cooperation agreements (Møre and Romsdal Health Trust, 2015).

Municipalities have sought to adjust to the payment scheme, for example, by developing 24-h housing services. However, scaling services to meet diverse patient needs and ensuring sufficient expertise has been challenging. Before the pandemic, compliance with the payment scheme was a major focus, often sparking disputes over discharge criteria. One key point of contention was whether municipalities should be fined for “overnight stays” when discharge summaries were missing. A hospital manager noted that municipalities presented strong arguments, “primarily based on the cooperation agreement, but above all on professional grounds”.

However, the scheme was seen as undermining the collaboration. A municipal leader noted that media could portray the situation as if the parties were imposing daily fines on each other. A clinical manager described it like this:

It resembles a game of blackmail, where we sit in our own trenches and throw stones at each other […] I don't really believe in punishment, but I do believe in dialogue and finding solutions that are best for the patient.

(Clinical manager)

During the pandemic, attention shifted from financial penalties to proactive collaboration. A hospital department manager noted that key personnel from both the hospital and the host municipality established early contact to address the potential severity of the pandemic. “Even before the first meeting, the municipality had begun planning for the reception of regular patients to free up hospital capacity for pandemic-related cases.”

A hospital adviser illustrated this shift in tone, recalling how collaboration meetings often began: “Now we are going to assist each other, how can we pull the load together?” Rather than focusing on disagreements, the pandemic emphasized joint problem-solving. As the GP put it: “It became very “to the point” […] from previously having a tendency to pick on each other when things didn’t work, to becoming very solution-oriented.”

According to Innes and Booher (2015), collaboration inherently involves conflict, as it brings together actors with different roles, values and interests. At the same time, conflicts through dialogue can lead to an exchange of arguments that contributes to new insights and supports coordinated action.

Before the pandemic, collaboration was hindered by conflicts related to the payment arrangement, reflecting an instrumental logic based on actions aimed at avoiding sanctions, for example, through discussions about payment terms to avoid daily fines. At the same time, the municipalities' efforts to expand services demonstrate a recognition of their responsibility for patients ready for discharge. However, this is not sufficient if the municipalities lack the necessary competence. The municipalities' need for sufficient information appears to have led to increased understanding within the hospital, not solely due to provisions in the cooperation agreement. This illustrates Innes and Booher's (2015) point that it is possible to develop new understanding when different actors meet and discuss even difficult issues. Although it is described as time-consuming to ensure that this understanding is integrated into hospital practice, the example shows how instrumental agreement conditions and exchanges of meaning aligned with communicative logic can support municipal needs and contribute to safer and more coordinated discharge processes.

During the pandemic, the payment arrangement did not seem to receive the same level of attention, as the focus shifted toward finding solutions to avoid hospital overload. This demonstrates Innes and Booher's (2015) point that although collaboration involves tensions, it is possible to set them aside. This depends on what the various actors aim to achieve through collaboration, whether to promote their own interests or to recognize the value of joint efforts toward mutual benefit. The pandemic appears to have contributed to an altruistic emphasis, in line with communicative logic. Our interpretation is that combating the pandemic created a collective benefit that could only be achieved through collaboration. This seems to have taken precedence over spending time discussing regulatory provisions and cooperation agreements.

### Managing scarce resources in the tension between process and outcome

The coordination reform outlined, at a general level, which tasks could be assigned to municipalities, provided they had sufficient resources. Since a clear division of responsibilities between levels of care is not feasible, the statutory agreement system must define the planned distribution of tasks and how health trust and municipalities will collaborate (Norwegian Ministry of Health and Care Services, 2009, p. 28). This objective is to be concretized in the cooperation agreements, based on mutual involvement and consensus (Møre and Romsdal Health Trust, 2015). To support decision-making, the actors have established a formal, hierarchical collaboration structure (Figure 1, p. 10) with detailed guidelines for representation and organization.

Informants emphasized that this development was both important and necessary. However, scaling up municipal services has been difficult, particularly given shorter hospital stays and limited resources. From the perspective of the GP, the coordination reform is experienced more as a transfer than a division of tasks: “When the specialist health service no longer has resources or can provide a service, the tasks are returned to the GPs”.

Most informants also highlighted the challenge of differing management structures and funding systems between health trusts and municipalities. A hospital manager noted that resource constraints are a frequent topic of discussion and often hinder progress in joint initiatives. A head of municipal affairs illustrated this tension: “It's fine to collaborate as long as it’s on our terms […] we would like to have resources, but the others prefer not to give up resources”.

Due to the actors' autonomy, joint decision-making has been difficult. A hospital leader expressed frustration with the overall collaborative committee, describing it as overly bureaucratic and inefficient, as decisions often require input from numerous clinic managers and municipalities, even on minor issues. Similarly, the dialogue meeting, bringing together top political and administrative leaders from the health trust and all 27 municipalities, was seen as not suited for presenting restructuring measures. Its large size limited meaningful anchoring, and as one mayor pointed out, failing to involve affected actors early can undermine even the best plans: “No matter how good the plan is, you get off to a bad start”.

Tensions over resources and a lack of involvement also surfaced during the pandemic, for example, in the distribution of infection control equipment and the implementation of municipal regulations without informing the health trust. However, such issues were less prominent in the overall collaboration. According to informants, the focus shifted toward task management and operational problem-solving.

To support effective decision-making, the health trust and municipalities established new informal arenas for collaboration (Figure 2, p. 11) and engaged in direct dialogue between relevant actors. A clinical manager described this shift like this: “It was so simple that we had to contact the municipality; we had to look at how many places we could use […] from me to the municipal chief physician, and it was not anchored in any agreement”. A head of municipal affairs emphasized that this approach was shaped by the urgency of the situation.

Finding practical solutions to something urgent, you can't wait until the next meeting. […] Maybe it will become more formal, it must be investigated, and it will be this and that. You are more dependent on getting frequent responses.

(Head of municipal affairs)

The GP highlighted how the need for rapid outcomes shaped decision-making and emphasized the trust placed in key actor:

As in war, there are orders from above where there is little discussion with those on the ground. It wasn't the GPs, it wasn't the users, but here we trusted, here we gave the hospital management, the municipal chief physician, the chief infectious disease physician, we trusted that whatever they decided was somehow okay.

(General practitioner)

In light of Innes and Booher (2015), our findings suggest that collaboration prior to the pandemic was largely process-orientated, while during the pandemic, it became more task-focused, addressing concrete challenges.

Before the pandemic, the actors appeared to attempt to facilitate democratic processes through the formal collaboration structure, in line with the guidelines for representation and mandate set out in the cooperation agreement. Nevertheless, outcomes such as consensus-based solutions were often hindered by the actors' autonomy and the challenge of anchoring joint measures across a health trust and 27 municipalities. Outcomes like deadlocked resource discussions reflect a central critique of the communicative planning ideology. One consequence, as noted by our informants, is that collaboration either comes to a halt or results in lowest common denominator outcomes (Innes and Booher, 2015, p. 4).

During the pandemic, the health trust and the municipalities focused on carrying out tasks efficiently. In light of the process–outcome dichotomy described by Innes and Booher (2015), this suggests that instrumental logic became more prominent, where achieving rapid results was prioritized over adhering to communicatively inspired process requirements set out in the cooperation agreement. We interpret this mindset as legitimizing informal collaborative dialogues, allowing for swift clarifications without lengthy investigations or the need for broad participation. Instrumental logic was also evident in how decision-making was informed by expert knowledge. As pointed out, those involved in the pandemic collaboration were entrusted to make the necessary decisions.

### Managing obligations in the tension between communication power versus state power

The coordination reform reflects an institutional shift in the relationship between national and local governance in Norway. Through democratic multi-level governance (Bache *et al.*, 2016; Helgøj and Aars, 2008; Rommetvedt *et al.*, 2014), national authorities establish the framework while simultaneously seeking to uphold the ideal of local self-government. This illustrates how capacity and coherence in health services are pursued through collaboration within a context of limited hierarchy (Indset *et al.*, 2012; Martinussen and Magnussen, 2009; Nylenna, 2024). A central challenge is determining how far the authorities can go in implementing system-level measures anchored in the patient pathway that may limit the autonomy of both health trusts and municipalities (Norwegian Ministry of Health and Care Services, 2009, p. 22).

To balance state control and local autonomy, the coordination reform employs both hierarchical and non-hierarchical instruments. Collaboration is mandated by law with minimum requirements, yet health trusts and municipalities retain the ability to adapt the guidelines in local cooperation agreements (Norwegian Ministry of Health and Care Services, 2011a, b). According to our informants, this governance logic has generated several dilemmas. As autonomous actors subject to different governance and funding mechanisms, it is considered demanding to secure professional and political support for measures involving financial risk and the allocation of scarce resources. This, in turn, has constrained the ability to reach consensus decisions in collaboration processes prior to the pandemic.

During the pandemic, these challenges became less pronounced, as a shared sense of responsibility and the ability to jointly address emerging tasks took precedence. We interpret this change both as a consequence of the shift in Norwegian governance logic toward a command-based response (Christensen *et al.*, 2023) and as a reaction to the urgent need to identify and implement measures that could prevent widespread infection and the overburdening of the healthcare system. Both national and local initiatives increasingly relied on expert knowledge to ensure professionally grounded solutions. Through ad hoc arrangements or within informal collaboration structures, the health trust and the municipalities demonstrated their capacity to act as equal partners, with an enhanced ability to coordinate and sustain local health services.

The collaborative behaviour observed before and during the pandemic illustrates Innes and Booher's (2015) argument that political power operates through communication. While power can be mobilized through state governance, it can also be generated through collective meaning-making, with both inhibitory and promoting effects on political goals. Prior to the pandemic, the coordination reform often contributed to discussion and conflicts, as political objectives created cross-pressure against the individual needs and priorities of the health trust and the municipalities. In contrast, during the pandemic, our findings indicate that the actors largely complied with the authorities' expectations. At the same time, there remained scope for a degree of autonomy, as the actors preferred to collaborate more informally among relevant participants.

Our findings demonstrate how the pandemic generated a collective sense of threat, shaping understanding across governance levels and influencing both national strategies and local implementation. Supported by what we interpret as a stronger degree of instrumental governance, this facilitated collaboration aligned with communicative principles such as equality, mutual appreciation and solutions that contributed to combating the pandemic. The way the COVID-19 virus was framed and placed on the agenda, with expectations directed toward concrete solutions, appears to have opened a productive space for action in the collaboration between the health trust and the municipalities.

## Conclusion

Collaborative strategies have become increasingly central to public health reforms, aiming to improve coordination and strengthen the capacity of health services. This study, situated in a Norwegian context, explores collaboration between a health trust and its associated municipalities. It demonstrates how multi-actor collaboration, in pursuit of political goals, encounters dilemmas that were handled differently before and during the COVID-19 pandemic. An overall finding is that the relationship between governance and collaboration is context-dependent, shaping the navigation of political dilemmas and conditioning their mutual interplay.

Drawing on Innes and Booher's (2015) framework, which links instrumental and communicative rationalities, this study interprets this variation through discursive tensions: community knowledge versus science, collaboration versus conflict, process versus outcome and communicative versus state power.

Framed by the governance logic of the coordination reform (Ministry of Health and Care Services, 2009), the findings reveal how power imbalances and conflicting interests challenged the implementation of legal mandates and financial incentives prior to the pandemic. During the pandemic, political instruments became less central, and actors recognized the necessity of a joint response. Informal dialogue among competent participants strengthened the collaboration's capacity for effective crisis management.

Before the pandemic, an instrumental understanding of knowledge reinforced the health trust's dominance. The interpretation of policy tools triggered conflicts, as efforts to ensure democratic involvement led to gridlocked discussions in the tension between state control and local adaptation. During the pandemic, professionals across levels were recognized as key contributors, and collaboration was seen as essential. The acute situation required more flexible, trust-based and informal collaboration, showing that actors retained some autonomy despite increased top-down governance aimed at reducing infection and securing healthcare capacity.

This shift illustrates how crises can restructure governance roles and foster a more productive interplay between instrumental control and outcomes shaped by communicative dialogue. Clear goals, mutual recognition of local perspectives and flexible structures, where relevant actors are entrusted with decision-making authority, can strengthen policy support and enable local mobilization. Health reforms should therefore aim to balance hierarchical control with communicative approaches to promote multi-actor collaboration.

While this study offers insights into collaborative patterns across different contexts, it has methodological and contextual limitations. Focusing on collaboration between one hospital trust and its associated municipalities limits generalizability, and causal relationships cannot be established. The chosen theoretical framework narrows the interpretive lens, and alternative perspectives may reveal other aspects of collaboration.

Future research should therefore explore how health policy instruments can balance instrumental governance with communicative, bottom-up approaches, both in depth and across diverse contexts. More nuanced understanding can also be achieved through examining collaboration through other theoretical lenses and by combining different methodological approaches. In particular, research should investigate how hybrid governance models can integrate formal structures with adaptive and more informal collaboration, both in routine settings and during crises. Greater attention should also be given to how relational dynamics and trust, including delegated decision-making authority, affect collaborative capacity across organizational levels and sectors and how communication shapes shared understanding and engagement. Such insights are crucial for designing resilient health systems capable of navigating both routine operations and future crises.
